# Renal disease and accidental falls: a review of published evidence

**DOI:** 10.1186/s12882-015-0173-7

**Published:** 2015-10-29

**Authors:** Pablo Jesús López-Soto, Alfredo De Giorgi, Elisa Senno, Ruana Tiseo, Annamaria Ferraresi, Cinzia Canella, María Aurora Rodríguez-Borrego, Roberto Manfredini, Fabio Fabbian

**Affiliations:** Department of Nursing, The Maimonides Institute for Biomedical Research in Cordoba, University of Córdoba, University Hospital Reina Sofía of Córdoba, Córdoba, Spain; Clinica Medica, Department of Medical Science, University of Ferrara, Ferrara, Italy; Department of Medicine, Azienda Ospedaliero-Universitaria (AOU) of Ferrara, Ferrara, Italy; U.O. Formazione e Aggiornamento, Azienda Ospedaliero-Universitaria (AOU) of Ferrara, Ferrara, Italy

**Keywords:** Aging, Chronic kidney disease, Frailty, Falls

## Abstract

**Background:**

The pathogenesis of falling is complex, and identification of risk factors may be essential for prevention. The relationship between renal disease and falls is unclear, and the goal of this study was to collect the available evidence and investigate the relationship between accidental falls and renal dysfunction.

**Methods:**

Electronic searches were performed in the MEDLINE, Scopus, Ovid SP and Web of Science databases to identify the appropriate literature. The themes used were: falls (combined in the title/abstract fall or falls or falling or faller* or fallen or slip* or trip* or (MeSH) accidental falls) and renal insufficiency (chronic or renal insufficiency or kidney diseases combined in title/abstract renal disease* or kidney disease* or renal insufficiency or kidney insufficiency or kidney failure or renal failure or MeSH renal insufficiency, chronic or renal insufficiency or kidney diseases). The incidence, risk factors, complications, and characteristics of the falls were analyzed.

**Results:**

Eight prospective cohorts including five cross-sectional studies, and one case–control study were identified. No randomized controlled studies were found. The incidence of falls in chronic kidney disease patients ranged between 1.18 and 1.60 fall/patient year. These were frequent in frail older adults on hemodialysis treatment. Falling relapses in the same group of patients caused serious consequences. Data on pre-end stage renal disease (ESRD) were scarce.

**Conclusions:**

The risk of falling appears to be common in patients with renal dysfunction especially in older adults undergoing hemodialysis. On the other hand, we could not find any conclusive data on pre-ESRD patients.

**Electronic supplementary material:**

The online version of this article (doi:10.1186/s12882-015-0173-7) contains supplementary material, which is available to authorized users.

## Background

Chronic kidney disease (CKD) is a common condition with significant medical, social, and economic burdens. It is commonly associated with several comorbidities especially in older adults [1, 2]. Cardiovascular and neurological diseases are the most important risk factors for falls [3–5], but CKD is also an intrinsic risk factor for falling [6].

An accidental fall is defined as “inadvertently coming to rest on the ground, floor or other lower level excluding intentional change in position to rest on the furniture, wall or other objects” [7]. The risk of falling increases with age, and one-third of people aged ≥65 years fall at least once per year [8]. In 2010, the cost of falls in the US was $30 billion [9].

Although falls are the consequence of a complex interaction between multiple risk factors [3], the coexistence of factors such as polypharmacy, comorbidities and changes in volume status suggests that patients with different degrees of CKD are more likely to fall than the general population.

Therefore, the aim of this study was to synthesize published research about accidental falls evaluating patients with renal failure. Furthermore, the quality of the scientific evidence obtained was also analyzed.

## Methods

### Design

A review of observational studies and randomized controlled trials provides a narrative synthesis and assessment of methodological quality of the included studies. The steps were searching, data extraction, assessing of quality, summarizing of finding and interpreting the results. This followed the Preferred Reporting Items for Systematic Reviews and Meta-Analyses (PRISMA) guideline [10, 11].

### Search methods

Electronic searches of the published literature were performed in MEDLINE, Scopus, Ovid SP and Web of Science. Each database had customized searches due to the different vocabulary search terms and interfaces of the search in each database. The Cochrane Database was checked for reviews on the topic but there are, to our knowledge, no previous studies. The keywords used were the Medical Subject Heading (MeSH) terms of “accidental falls”, “chronic”, “renal insufficiency” and “kidney diseases”. No age limits, restriction of language or date of publication were used.

The search strategy combined two search themes with the Boolean operator “and”. The first theme was falls combined in the title/abstract fall or falls or falling or faller* or fallen or slip* or trip* or (MeSH) accidental falls. The second theme was renal insufficiency, chronic or renal insufficiency or kidney diseases combined in title/abstract renal disease* or kidney disease* or renal insufficiency or kidney insufficiency or kidney failure or renal failure or MeSH renal insufficiency, chronic or renal insufficiency or kidney diseases.

### Inclusion and exclusion criteria

We included all studies with following criteria: (i) observational studies (cross-sectional, case–control, and cohort studies) or randomized controlled studies; (ii) studies including CKD adults and older adults (both pre-end-stage renal disease (ESRD) and ESRD); (iii) studies analyzing CKD patients with an increased propensity to fall.

We excluded: (i) root cause analysis studies or reviews or studies in dissertations and editorials; (ii) studies that were the end-point of bone evaluation.

### Search outcome

The search terms and database searches were determined by two reviewers (E.S., P.J.L). They reached a consensus with respect to databases and search terms. These databases were chosen for their large coverage on the topic as well as the authors’ previous experience with these databases. Between 20/10/2014 and 27/10/2014, each reviewer independently performed the electronic searches finding the same results in each database with the terms described above. They found a total of 13745 records.

**Fig. 1:**
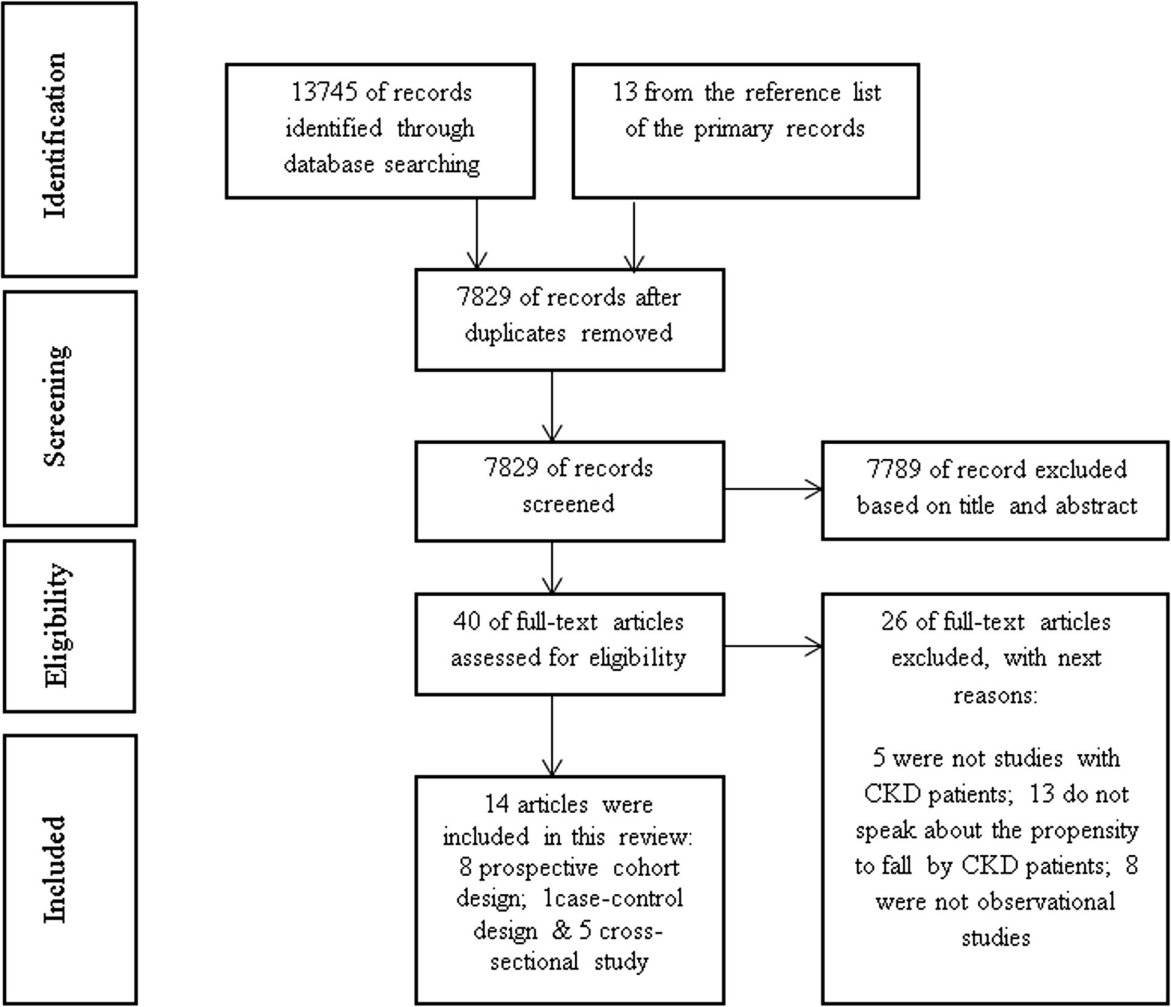
PRISMA flow diagram of the screening process

According to the Moher et al. [11] recommendations, all details of the search process are shown in a PRISMA flow chart (Fig. 1). Researchers independently reviewed titles and abstract, and selected articles addressing the relationship between falls and CKD patients. They removed duplicate articles. At this step, 29 relevant titles were selected. On the second step, after reading the full article, we selected 14 articles that met with the a priori established inclusion and exclusion criteria. Disagreements in selected articles were resolved by discussion and consensus. Moreover, further relevant articles were added from the reference list of the primary articles.

### Quality appraisal of the publication

The methodological quality of the articles was assessed by the same researchers who performed the search (E.S., P.J.L.). We assessed the articles using the Strengthening the Reporting of Observational studies in epidemiology (STROBE) checklist because there is no accepted gold standard for evaluating the methodological quality of selected articles (Version 4 published in October/November 2007 [12]). We did not intend to use this tool to determine methodological quality, but merely to use this checklist with 22 recommendations for the design of all selected studies (cross-sectional, case–control, and cohort studies) (Additional file 1).

In addition, to assess the quality of the same studies, we also applied the GRADE (Grading of Recommendations Assessment, Development and Evaluation) approach to address the following five domains: type of evidence, quality, consistency, directness and effect size [13].

### Data abstraction

Data were extracted from each study according to the events of fall, main outcomes, fall risk factors, and methodology [14, 15] as well as the setting and sample (Table 1) and their characteristics (age, sex, and cause of renal disease) (Tables 2, 3 and 4).

**Table 1:** Parameters summarizing the main studies dealing with falls in renal dysfunction patients. Author, number of patients and events, setting, study design, risk factors and main outcomes are reported

Author (year)	Sample (events)	Country & setting	Type of study (study period)	Fall risk factors main outcomes	Checklist (Reporting)
Roberts (2003)	47 patients	UK	Cross-sectional study	There were significantly more patients reporting falls and/or syncope in elderly patients who had post-dialysis orthostatic hypotension.	15
	(13)	1 Hospital HD unit	(12 months)		
Cook (2005)	135 patients	Canada	Cross-sectional study	There was no significant difference in the incidence of falls among “young-old” and “old-old” group in either gender.	14
	(37)	2 Outpatient HD unit	(12 months)		
Desmet (2005)	308 patients	Belgium	Prospective cohort study	Older age, diabetes, failed walking test, intake of an antidepressant and high number of oral prescribed drugs were identified as independent predictors of falling	18
	(56)	7 In-center HD units	(8 weeks)		
Cook (2006)	169 patients	Canada	Prospective cohort study	Male gender, a history falls, low mean pre-dialysis SBP, and higher comorbidity were important risk factors for falls	22
	(305 falls over a median of 468 days)	1 Outpatient HD unit	(12 months)		
Angalakuditi (2007)	635 cases & 1270 controls	USA	Retrospective case–control study	Increased likelihood of experiencing an in-hospital fall occurred with dementia, pneumonia, gastrointestinal disease and diabetes, as well as taking antidepressants and anticonvulsants.	21
	(Falls determine the cases: 635 cases)	1 University medical center	(5 years & 6months)		
Roberts (2007)	78 patients	UK	Prospective cohort study	Older patients fell more than younger patients. There was no relationship between incidence of falls and the routine blood pressures nor with hemoglobin concentration or number of medications.	8
	(14)	1 Hospital HD unit	(6 months)		
Li (2008)	162 patients	Canada	Prospective cohort study	Falls were associated with double risk of death. Risk of death increased with 1-year in dialysis or 1-year in age or with changes in hemoglobin, serum albumin and the calcium-phosphate product	18
	(305)	1 Outpatient HD unit	(12 months)		
Boudville (2010)	25 patients (9 with 25 OHD ≤50 nmol/l; 16 with 25 OHD > 50 nmol/l	Australia	Cross-sectional study	Suboptimal levels of 25 OHD may contribute to an increased risk of falls. Although, not significant, there were more falls in patients with suboptimal levels of 25 OHD.	20
	Not determine incidence of falls	1 Outpatient HD unit	(No data)		
Abdel-Rahman (2011)	76 patients	USA	Prospective cohort study	Female gender was a significant predictor of falls. Compared to ‘non-fallers’, ‘fallers’ had higher risk of death, nursing home admission, and increase in number and duration of hospital.	18
	(20)	2 Outpatient HD unit	(12 months)		
Rossier (2012)	84 patients	Switzerland	Prospective cohort study	POMA score along with age, a past history of falls, malnutrition and depression, were associated with severe falls.	19
	(31 severe falls)	1 Hospital HD unit	(3 years)		
Galvão (2013)	64 patients	Brasil	Cross-sectional study	No correlation between PTH serum levels and FES-I. Higher tendency to fall among the patients who presented low calcitriol serum levels. FES-I can be capable of discerning falling from no-falling patients in HD	19
	Not determine incidence of falls	1 Outpatient HD unit	(No data)		
McAdams-DeMarco (2013)	95 patients	USA	Prospective cohort study	Fragility is an independent fall risk factor adjusting for age, sex, race, comorbidity, disability, number of medications, marital status and education. No difference between younger and older adults was seen.	14
	(70)	1 Outpatient HD unit	(15 months)		
Kutner (2014)	762 patients	USA	Cross-sectional study	Frail patients were over twice as likely to report falls. Patients with depression (CES-D >18) and/or prescribed antidepressants were over 80 % more likely to be faller than were patients with no depression (CES-D < 18) and no prescribed antidepressants.	17
	(671)	8 Outpatient HD unit	(12 months)		
Rothenbacher (2014)	1385 patients	Germany	Prospective cohort study	25 OHD serum level were associated with risk of first fall. Calcium levels modified the effect. No association existed between chronic kidney disease and risk of first fall.	17
	Not determine incidence of falls	Community	(12 months)

**Table 2:** Age, sex, primary renal disease and comorbidity of patients enrolled in prospective studies

	Mc-Adams [23]	Rossier (2012 )	Rothenbacher [19]	Abdel-Rahman [23]	Li [21]	Roberts [17]	Cook [20]	Desmet [27]
Age (mean ± SD) years	60.5 ± 12.6	69.5	75.6	62.4 ± 6.1	74.7 ± 6.1	58	74.7 ± 6.1	70.9
Male (%)	53.7	67	57.2	61.8	57	65.4	57	N/A
Female (%)	46.3	33	42.8	38.2	43	34.6	43	N/A
Renal diagnosis								
Hypertension (%)	N/A	29.8	N/A	N/A		N/A		N/A
Diabetes mellitus (%)		29.8			27		27	
Glomerulonefritis (%)		13.1			12		12	
Hypertensive/renovascular (%)		N/A			28		28	
Other (%)		N/A			28		28	
Unknown (%)		N/A			5		5	

N/A = not applicable

**Table 3:** Age, sex, primary renal disease and comorbidity of patients enrolled in cross-sectional studies

	Cook [26]	Boudville [27]	Roberts [24]	Galvao [28]	Kutner [25]
Age (mean ± SD) years	74.9 ± 6.2	69.5 ± 12.1	78.2 ± 5.3	44.2 ± 14.8	57.1
Male (%)	61	80	49	73.4	59.2
Female (%)	39	20	51	26.6	40.8
Renal diagnosis					
Diabetes mellitus (%)	N/A	24	N/A	N/A	36.9
Glomerulonefritis (%)	N/A	20	10	N/A	N/A
Hypertension (%)	N/A	20	31	N/A	34.9
Polycystic kidney disease (%)	N/A	4	6	N/A	N/A
Obstructive nephropathy (%)	N/A	N/A	12	N/A	N/A
Nephrocalcinosis (%)	N/A	N/A	4	N/A	N/A
Chronic pyelonephritis (%)	N/A	N/A	2	N/A	N/A
Analgesic nephropathy (%)	N/A	N/A	2	N/A	N/A
Acute illness (%)	N/A	N/A	2	N/A	N/A
Other (%)	N/A	32	N/A	N/A	N/A
Unknown (%)	N/A	N/A	25.5	N/A	N/A

N/A = not applicable

**Table 4:** Age, sex and primary renal disease of patients enrolled in case–control study

Angalakuditi et al. [29]	Case	Control
Age (mean ± SD)	68.5 ± 15.3	69.1 ± 15.3
Sex		
Male (%)	46.3	N/A
Female (%)	53.7	N/A
Renal diagnosis	N/A	N/A

N/A = not applicable

### Synthesis

There were 14 studies that met the inclusion criteria. We used a narrative approach due to the heterogeneity of the selected studies regarding methodology and difference in their designs and outcomes [15].

## Results

The 14 relevant studies were all observational even if there were methodological differences. Eight of the fourteen studies had a prospective cohort design, five were cross-sectional and one was a case–control. No randomized controlled studies were found.

### Prospective cohort studies

Four of the eight prospective studies were performed in Europe [16–19], while the other four were in North America (two in Canada [20, 21] and two in the USA [22, 23]. All studies were performed in hemodialysis (HD) units. The population of the relevant prospective studies varied between 76 [24] and 1385 subjects [19] with a total sample of 2357 patients. The mean age ranged between 58 [17] to 75.6 [19] years, however we identified subgroups with lower mean ages (47.1 years in patients under 65) [22] and higher mean age (75.8 years in patients over 65) [16].

#### Incidence

Five [16–18, 20, 22] studies provided data on the incidence of falls. Roberts et al. [17] reported an incidence of 1.76 falls/patient year in subjects over 65 and of 0.13 falls/patient year in younger ones. Abdel-Rahman et al. [22] found an incidence of 1.54 falls/patient year in subjects over 65. Cook et al. [20] reported an incidence of 1.60 falls/patient year, but when one individual with 48 falls was excluded, the incidence was reduced to 1.36. Only three articles reported data on the incidence of severe falls [16, 18, 22]. Desmet et al. [16] found an incidence of 0.37 falls/patient year for events requiring medical care, while Rossier et al. [18] and Cook et al. [20] described incidences of 0.22 and 0.20 falls/patient year, respectively.

#### Risk factors

Five [18–20, 22, 23] of the relevant prospective studies identified several risk factors for falls. Age over 65 years was a risk factor in three studies [16–18]. Gender was evaluated in three studies [20, 22, 23], but the results were conflicting. The relationship between falling and male gender was reported by Cook et al. [20], whilst Abdel-Rahman et al. [22] and McAdams-DeMarco et al. [23] demonstrated the risk for falling was higher in female patients. Other fall risk factors included a history of falls [18, 20], depression and therapy with antidepressants [16, 18], number of prescribed oral drugs [16], frailty [23], failed walking test [16], malnutrition [18], comorbidity [20], diabetes [16], and high school education [23].

Two studies estimated the proportion of patients remaining free of falls using Kaplan-Meier analysis [18, 19]. Rossier et al. [18] calculated that after a mean follow-up of 465 days, 89.1 % of patients < 65 years were free of falls. Those between 65 and 75 were 57.2 % free of falls, but only 30 % in those over 75. Rothenbacher et al. [19] estimated a risk of first fall that was associated with CKD but found no statistically significant relationship. Li et al. [21] found that a history of falls and serum albumin were independent risk factors for death associated with accidental fall.

#### Complication of falls

Data related to falls and complications of falls were reported in three studies [16, 20, 22]. Desmet et al. [16] found that the percentage of fall-related fractures was 3.9 %. Similar results were found by Cook et al. [20] (4 % of fall-related fractures), however in their study, six patients died and 26 were hospitalized as a direct result of the fall. Abdel-Rahman et al. [22], found 20 deaths (0.151 deaths per patient-year), 14 nursing home admissions and 219 hospitalizations over 2 years. They calculated higher rates for the risk of death (2.13-fold increase), risk of nursing (3.5-fold increase), and risk and number and duration of hospitalizations (2-fold increase) in ‘fallers’ than ‘non-fallers’. They also found higher number of hospitalizations when comparing ‘recurrent fallers’ vs. ‘non-fallers’.

#### Characteristics of falls

The location of falls was evaluated in two studies [16, 20]. Desmet et al. [16] reported a higher number of falls occurring at home (82 %) followed by public sites (7 %) and other (9 %) or unknown locations (2 %). Cook et al. [20] found different percentages between indoor (69 %) and outdoor falls (31 %). In the latter study, the authors evaluated the modality of the fall with walking (57 %) being the most common activity followed by standing from the seated position (31 %) and trying to rise from a lying position (12 %). In both studies, the time of fall was analyzed. Desmet et al. [16] found a higher number of first falls within 22 h from a HD session; a similar frequency was seen with falls on dialysis and non-dialysis days [20]. However, this group showed a higher number after (73 %) than before (27 %) dialysis.

### Cross-sectional studies

Five relevant cross-sectional studies were available [25–28]. All studied outpatient HD units. The five studies were conducted in different countries. The total population of the cross-sectional studies ranged from 25 to 762 subjects with a final total sample of 1033 patients of various characteristics. The mean age of the population ranged from 44.2 to 78.2 years.

#### Average number of reported falls

Two out of the five studies determined the number of falls [24, 26], and one reported the incidence of falls. Roberts et al. [17] reported 13 falls in a population of 47 patients; Cook et al. [20] calculated 37 falls over 12 months, and 21 falls in the prior 12 months in a population of 135 HD patients. Meanwhile, 671 falls were reported by Kutner et al. [25] in 762 patients for an incidence of 0.88 falls/patient year. In this study, no differences were found between amputees and non-amputees.

#### Risk factors

Kutner et al. [25], reported that frailty was independently associated with higher risk of falls versus non-frail patients. Age increased the risk of fall, but elderly patients with good cognitive function scores had lower risks of falling. No significant risk factors for falls were found in the other four relevant cross-sectional studies in which age [26], sex [27], postural hypotension [24], levels of 25-hydroxyvitamin D (25-OH-D) [27] and other validated tools [27, 28] were considered as predictors of falls.

#### Complications of fall

Only Kutner et al. [25] calculated data about complications of falls—they described fractures in 11.2 % of fallers (216 patients). The most common were upper or lower limb fractures followed by two hip fractures and one forehead injury; 71 % of patients were hospitalized.

### Case–control design

One relevant case–control study was selected [29]. This study evaluated the association between comorbidities and drug use with risk of the in-hospital falls in CKD patients. Falls were recorded in 635 out of 1905. The majority of falls had no complications (77.3 % of cases), but abrasion was described in 5.4 % of cases, pain in 2.8 %, laceration in 2.2 %, blood loss in 1.9 % and fractures in eight (1.3 %). A significant percentage of subjects were discharged to nursing homes (27.7 %), rehabilitation units (18.4 %), short or intermediate skilled-care nursing facilities (5.8 %), and to other hospitals (2.0 %). Unfortunately, 7.7 % of subjects died while hospitalized.

The majority of patients fell from the bed (39.6 %), and a 33 % fell while ambulating. Other episodes include falls in the bathroom (16.0 %), from a chair (9.1 %), or falls that were not witnessed (found on the floor episodes (9.1 %)). The time of fall in relation to length of stay determined that the majority of falls (57.6 %) occurred within the first week of hospitalization.

#### Risk factors

Comorbidities such as dementia, pneumonia, cardiac arrhythmias, gastrointestinal disease, and diabetes mellitus were risk factors for falling as well as the use of antidepressants and anticonvulsants.

### Methodological quality

Using the STROBE checklist we assessed the quality of reporting of observational studies. This checklist is purely informative because it is not possible to determine the quality with this tool—only the recommendations are discussed. For this reason, the GRADE approach is used to assess the quality of the evidence. Due to the heterogeneity of the variables evaluated in different studies, we could only analyze age, frailty, previous falls and polypharmacy (Table 5).

**Table 5:** Quality of the risk factors for falling upon comparing fallers to non-fallers according the GRADE approach (based on our systematic review). Variables evaluated in different studies were heterogeneous, therefore only age, frailty, previous falls and polypharmacy could be analyzed

Outcome	No. of studies	Design	No quality	Inconsistency	Indirectness	No effect size	Quality
Age	4 studies (700 subjects)	Prospective cohort studies	Very serious	Serious	Serious	Very serious	Very low
Frailty	2 studies define this specifically, four others are related (857 subjects)	Prospective cohort and cross sectional studies	No	Little or no	No	No	Low
Previous falls	3 studies (405 subjects)	Prospective cohort studies	Little or no	No	Little or no	No	Low
Polypharmacy	3 studies (3598 total subjects; 1905 in case and control study)	Prospective cohort and retrospective case–control studies	Serious	Serious	Very serious	Serious	Very low

On this basis, not all studies could be considered of acceptable quality. The quality of evidence for age and polypharmacy was very low. The data quality regarding falls and previous falls was categorized into a higher level but was still relatively low.

## Discussion

This review presents an overview of the available evidence dealing with the relationship between falling and CKD. Despite the important and comprehensive searches on this topic, we found only fourteen articles that met the established inclusion criteria.

The majority of studies were of moderate quality. In 8 out of 14, the authors designed a cohort investigation—only a few studies had a poor methodological design. We found that the incidence of falls in CKD patients ranged between 1.18 [16] and 1.60 [20] falls/patient year; falling was a result of relapsing in the same group of patients especially in older and frail patients.

Although the mean age of the selected studies ranged between 44 and 78 years, most data was related to older adults [30].

### Risk factors of falls

Many different etiological factors might cause falling. Knowledge of these causes could facilitate the development of preventive measures.

Age is the main risk factor for falling in the general population [7]; up to half of people over 65 experience a fall every year [30]. Although not uniformly evaluated, age was significantly related to falls in CKD patients. Falls were more common in people over 65 years than younger subjects [16, 17]. On the other hand, McAdams-DeMarco et al. [23] could not detect any difference in the frequency of falls in those under 65 and 65 and older (25.9 %, vs 29.3 %) over a median period of 6.7 month.

In this study after adjusting for age, sex, race, comorbidity, disability, number of medications, marital status, and education, frailty independently predicted a higher number of falls [23]. There were no sex-based differences in the risk of falling. Some studies have shown that women have an increased risk of falling [31], while others have shown similar data on men [32]. The higher prevalence of falls in women was associated with strength reduction or decrease in bone mineral density [20, 33, 34].

Frailty is an important risk factor for falls, however frailty has not been universally defined, and several conceptual models to define it have been used [35–37]. Frailty can be considered to be a syndrome of impaired homeostasis and resistance to stresses that leads to an individual’s increased vulnerability and risk of adverse outcomes [38]. The Fried model is the most commonly used, and a person is considered to be frail when he/she develops three or more of the following symptoms: weight loss, exhaustion, loss of grip strength, decreased gait or low physical activity [36]. Only two studies [23, 25] reported a relationship between frailty and falling in CKD patients, and the association was strong. The lack of data regarding this relationship could be ascribed to the heterogeneity in the definitions used or to underestimation of the problem by nephrologists. In the general population, there is a widespread range in the prevalence of frailty ranging from 33 to 88 % [39].

Nevertheless, there are several studies suggesting that frailty could be a strong risk factor for falling. In fact, Rossier et al. [18] reported a relationship between falls and malnutrition in CKD patients. Malnutrition is related to sarcopenia, which in turn is related to frailty [40] and deficiency in vitamin D, antioxidants and oligoelements as well as proteins associated with osteoporosis, disability and sarcopenia. Moreover, Li et al. [21] found suboptimal serum albumin levels as a risk factor for fall in CKD patients.

Serum 25-OH-D levels were related to falls [41] and death [42]. The 25-OH-D levels in CKD patients are generally lower than in the general population [19, 28]. Boudville et al. [25] showed a relationship between suboptimal 25-OH-D levels and reduced quadricep muscle strength and consequently, an increased risk of falls. No relationship was detected with 1,25-OH-D levels demonstrating the greater influence of the active metabolite 25-OH-D on muscle strength versus 1,25-OH-D. Rothenbacher et al. [19] analyzed a group of patients with CKD and suboptimal serum calcium levels. They found an inverse correlation between serum 25-OH-D and the risk of first fall. These data show that serum 25-OH-D levels, especially when serum calcium level are suboptimal, may be considered a risk factor for falling in CKD patients independent of the degree of renal dysfunction. This is mainly due to the loss of muscle strength.

The use of different tools or tests such as the inability to perform a 10-min walking test (walk for ten minutes without any help) to determine the risk of falls has been reported in the study of Desmet et al. [16]. Different studies [11, 12] analyzed the utility of the POMA test (Mobility Assessment-Performance Oriented) because it easily establishes balance and walking ability. Galvao et al. [28] evaluated the capacity of the POMA score to determine the risk of falling in CKD patients.

In community-dwelling older people, there is a strong association between the risk of falling and having had a previous fall. The association is even stronger when there is more than one previous fall [4]. Similar results have been found in CKD patients by Rossier et al. [18], Cook et al. [20] and Li et al. [21].

Two prospective cohort studies [16, 23] reported that multi-therapy including selective serotonin reuptake inhibitors are a risk factor for falls in CKD patients especially those over 60 [29, 43].

The relationship between diabetes mellitus and falling could be explained on the basis of its complications such as impaired vision due to retinopathy and peripheral neuropathy. In a cross-sectional study conducted in the general population, the risk of falls in patients with poorly controlled diabetes was high [44].

The majority of studies enrolled HD patients, and according to many authors, HD is a risk factor for falling because large amounts of intravascular volume are removed causing an electrolyte imbalance and post-dialysis hypotension. However, Roberts et al. [17] did not detect any relationship between changes in blood pressure before and after the HD session and falls. Moreover no relationship was found when patients were categorized by age—thus, patients over 70 might be particularly susceptible to changes in blood pressure.

### Consequences of falls

The incidence of serious falls in CKD patients ranged between 0.20 and 0.37 falls/patient year. Abrasion, laceration and distortion were the most frequent consequences [20]; fractures ranged between 4 % and 11.2 % [20, 25]. Kutner et al. [25] underlined that patients who suffered severe fractures recovered their independence after the fall in 71 % of cases. Moreover, Cook et al. [20] reported that 16 % of falls required hospitalization, and 4 % of falls caused the patient’s death.

CKD patients have many complications due to falls [7, 30]. One year after the fall, 25 % of older people will die, 76 % will have limited mobility, 50 % will not be able to perform activities of daily living, and 22 % would be admitted to a nursing home [29]. In addition to the physical consequences reported, Desmet et al. [16] stressed the importance of post-fall syndrome including fear of falling, which encourages the patient not to rely on walking. This causes a decrease in physical activity and creates a vicious cycle that further reduces physical activity and muscle mass.

### Limitations

This review focused specifically on falls in CKD patients. On this premise, an electronic exhaustive search without language limitations was conducted. However, the number of selected studies was limited, and we cannot exclude the fact that this search method might not be fully comprehensive; however, relevant information has been extracted from the selected studies. We could not find a single randomized controlled study.

Although there is insufficient evidence to determine the methodological quality of the selected articles, we used the STROBE checklist to assess the reporting and the GRADE approach to assess the quality of evidence.

## Conclusions

We concluded there are not many studies with high methodological quality despite their publication in peer-reviewed journals. Thus, this review summarizes the current knowledge about falls in CKD patients.

To the best of our knowledge, data analyzing the impact of falls in CKD patients are scarce. The main result of this review is that falling is common in hemodialysis patients and nothing is currently known about falling in uraemic patients treated with transplantation or peritoneal dialysis or in subjects with pre-ESRD. This is a real problem for the health system because the number of elderly people with advanced renal failure is continuously increasing [28].

Given the multifactorial nature of falls, the identification of risk factors is essential for preventing such events. Frailty remains the most significant risk factor for falls, and its relevance is due to several factors including malnutrition, which is common in uremia. Malnutrition also causes disability and sarcopenia—factors that increase the risk of falling. Furthermore, the presence of CKD is associated with a decrease in the muscle strength in subjects who are elderly, have different comorbidities and/or need polypharmacy.

Despite the relevant information extracted here, more studies are needed especially randomized controlled studies to calculate precisely the incidence of falls in CKD population, the possible consequences of these falls, and a better definition of relevant risk factors. There should be a particular emphasis on frailty. Special attention must be placed on the elderly who are treated by HD—this appears to be a risk factor. Moreover a study should be designed to define if HD or peritoneal dialysis negatively impacts the risk of falls in older adults who need to start renal replacement therapy. This should address not only the underlying disease but also the effect of treatment. It might be worth also evaluating different risk factors for falls such as vision problems, substance abuse, and nocturia especially in pre-ESRD patients.

All of this information would provide further evidence to develop comprehensive and adjusted fall-prevention measures. This would reduce the economic and social burden for both families and health systems.

## Additional file

Additional file 1:
**Checklist of items to include when reporting a systematic review (with or without meta-analysis) PRISMA 2009 Flow Diagram.** (DOC 82 kb)

## Abbreviations

CKD: Chronic kidney disease
MeSH: Medical Subject Heading
PRISMA: Preferred Reporting Items for Systematic Reviews and Meta-Analyses
STROBE: Strengthening the Reporting of Observational
HD: Hemodialysis
25-OH-D: 25-Hydroxy Vitamin D
POMA: Mobility Assessment-Performance Oriented
