# Supplementary material for: Renal disease and accidental falls: a review of published evidence
Source: BMC Nephrol. 2015 Oct 29;16:176. doi: 10.1186/s12882-015-0173-7 (PMC4625452; doi:10.1186/s12882-015-0173-7)
Supplement: Additional file 1: — Checklist of items to include when reporting a systematic review (with or without meta-analysis) PRISMA 2009 Flow Diagram. (DOC 82 kb) [file 12882_2015_173_MOESM1_ESM.doc]

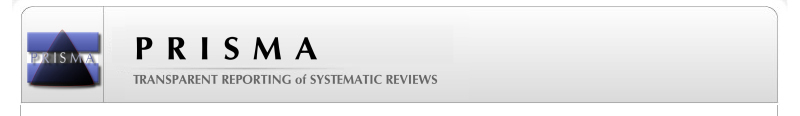
**PRISMA 2009 Flow Diagram**

**Screening**

**Included**

**Eligibility**

**Identification**

Records identified through database searching
(n = 13732 )

Additional records identified through other sources
(n = 13745 )

Records after duplicates removed
(n = 7829 )

Records screened
(n = 7829 )

Records excluded
(n = 7789 )

Full-text articles assessed for eligibility
(n = 40 )

Full-text articles excluded, with reasons
(n = 26 )

Studies included in qualitative synthesis
(n = 14 )
